# Comparative Transcriptomic Analyses of Antibiotic-Treated and Normally Reared *Bactrocera dorsalis* Reveals a Possible Gut Self-Immunity Mechanism

**DOI:** 10.3389/fcell.2021.647604

**Published:** 2021-09-21

**Authors:** Jiajin Fu, Lingyu Zeng, Linyu Zheng, Zhenzhen Bai, Zhihong Li, Lijun Liu

**Affiliations:** College of Plant Protection, China Agricultural University, Beijing, China

**Keywords:** *Bactrocera dorsalis*, intestinal bacteria, immunity, transcriptome, antibiotic treatment, *PGRP-SC2*

## Abstract

*Bactrocera dorsalis* (Hendel) is a notorious agricultural pest worldwide, and its prevention and control have been widely studied. Bacteria in the midgut of *B. dorsalis* help improve host insecticide resistance and environmental adaption, regulate growth and development, and affect male mating selection, among other functions. Insects have an effective gut defense system that maintains self-immunity and the balance among microorganisms in the gut, in addition to stabilizing the diversity among the gut symbiotic bacteria. However, the detailed regulatory mechanisms governing the gut bacteria and self-immunity are still unclear in oriental fruit flies. In this study, the diversity of the gut symbiotic bacteria in *B. dorsalis* was altered by feeding host fruit flies antibiotics, and the function of the gut bacteria was predicted. Then, a database of the intestinal transcriptome of the host fruit fly was established and analyzed using the Illumina HiSeq Platform. The gut bacteria shifted from Gram negative to Gram positive after antibiotic feeding. Antibiotics lead to a reduction in gut bacteria, particularly Gram-positive bacteria, which ultimately reduced the reproduction of the host flies. Ten immunity-related genes that were differentially expressed in the response to intestinal bacterial community changes were selected for qRT-PCR validation. Peptidoglycan-recognition protein SC2 gene (*PGRP-SC2)* was one of the 10 immunity-related genes analyzed. The differential expression of *PGRP-SC2* was the most significant, which confirms that *PGRP-SC2* may affect immunity of *B. dorsalis* toward gut bacteria.

## Introduction

*Bactrocera dorsalis*, also known as the oriental fruit fly, belongs to the Diptera (Tephritidae) family. As a member of the *Bactrocera* genus, it is listed as a quarantine pest in China. It has a wide host range and can damage more than 250 host plants (Verghese et al., 2012). It has been reported that the damage caused by oriental fruit flies can reach 100% of unprotected orchards, which causes huge losses to local agriculture and forestry (Zhang et al., 2010). Therefore, exploring effective control techniques for this notorious pest is of great significance.

Many studies have shown that different symbionts play an important role in the development of insects due to the different metabolic capacities of the host at different developmental stages (e.g., Lindow and Brandl, 2003; Knief et al., 2011; Li et al., 2016). Among the bacteria in the insect gut, “resident bacteria” are mainly involved in immune function and decomposing toxins. Previous results have shown that intestinal bacteria can help host insects resist pathogens (Ning et al., 2009; Bonnay et al., 2013). Similarly, the intestinal tract of *B. dorsalis* also contains a large number of symbiotic bacteria, which are mainly Acetobacaceae, Lactobacillaceae, Enterobacteriaceae, Cystosporaceae, and Brevibacterium (e.g., Naaz et al., 2016; Bai et al., 2018; Noman et al., 2020). The number of commensal bacterial communities could be significantly reduced under antibiotic treatment (Yao et al., 2016). Intestinal bacteria could also enhance the ability of host oriental fruit flies to degrade pesticides (Damodaram et al., 2016; Yao et al., 2016; Cheng et al., 2017). In summary, intestinal symbiotic bacteria may play an important role in the development, health and reproduction of *B. dorsalis*, and studying the function of intestinal symbiotic bacteria will be helpful in the development of new control strategies for insects belonging to the *Bactrocera* genus (Lauzon and Prokopy, 2013).

Insect immunity is made up of innate immunity and acquired immunity. Intestinal immunity is a kind of innate immunity. The insect immune system has no immune cells, proteins, or specific antigen-antibody responses like those in higher animals (Zasloff, 2002), but it can activate immune effectors via resident intestinal bacteria or toxins and then participate in the corresponding cellular and humoral immunity to resist the infection of foreign pathogens (Bonnay et al., 2013). For insects, an effective gut immune defense system takes a long time to build up to maintain the immune balance within the body, among gut microbes, and between the body and gut microbes (Sansonetti, 2004; Artis, 2008). Physical defenses, the immune deficiency (Imd) pathway, dual oxidase–reactive oxygen species (Duox-ROS), the Janus kinase-signal transducer and activator of transcription (JAK/STAT) pathways, and the intestinal symbiotic flora have been reported to be the main regulatory mechanisms of microbial homeostasis in the insect gut (Bai et al., 2018). However, there has been little research on the intestinal immune mechanism of *B. dorsalis* thus far. Studies on the cascade of immune-related pathways and the relationship among immune-related genes are also still lacking in this notorious pest. Many researchers are most interested in what the most important regulatory pathways and the most important key genes in oriental fruit flies are.

Our previous study showed that the gut bacterial diversity in *B. dorsalis* and *Zeugodacus tau* can be changed by antibiotic feeding, which resulted in the suppression of ovary development; in particular, the ovary of *Z. tau* was totally suppressed and could not produce eggs when fed a diet with antibiotics (Bai et al., 2018; Noman et al., 2020). How the gut bacteria maintain balance and how they regulate the growth, development, and reproduction of host flies are the scientific questions we are working on now. In this study, female and male *B. dorsalis* adults before and after antibiotic treatment were sequenced, and the differentially expressed genes (DEGs) were analyzed by RNA-Seq. Immune-related genes and KEGG pathways were assessed, and their functions in the intestinal immune process of *B. dorsalis* were discussed. This study will lay a theoretical foundation for the study of the invasion mechanism and biological control strategy of *B. dorsalis*.

## Materials and Methods

### Insects

*Bactrocera dorsalis* specimens were collected from Yunnan Province and reared in an artificial climate incubator for more than 20 generations. The rearing conditions were 25°C, 70% humidity, and 10 D:14 L (10 h dark and 14 h light). The fruit flies were reared using artificial food following the method described by Bai et al. (2018).

### Antibiotic Feeding Treatment

Antibiotics [tetracycline 120 μg/ml, streptomycin 400 μg/ml, and ampicillin 400 μg/ml at a ratio of 3:10:10 (Bai et al., 2018)] were added to the artificial food. Both larvae and adults were treated with equal proportions of antibiotics. Thousands of eggs were transferred onto solid food containing antibiotics and normal solid feed and then reared in an incubator. The third-instar larvae were picked out and then put onto sterilized moist sand while waiting for pupation and eclosion.

### Gut Bacterial Analysis

The methods used for fruit fly treatment and gut bacterial diversity analysis were from Bai et al. (2018). Bacterial diversity was analyzed via high-throughput sequencing of the V3–V4 variable region of the 16S rRNA gene, and then bacterial function prediction was performed via PICRUSt software.

### Growth and Reproduction Parameters Measurement

Adult weight and survival rate of 10-day-old adults (10 days post eclosion) were chosen as two growth parameters. The number of pupae and 10-day-old adults were recorded and used for survival rate calculation. Four adults were directly weighed by an electronic balance. The data were recorded, and the average weight was calculated. The preoviposition period, continuous spawning period and egg number were measured to represent reproductive ability. The *B. dorsalis* adults were observed at a specific time every day, and when they began and finished producing eggs was recorded. Egg amounts from five pairs of fruit flies (five female adults and five male adults) were recorded every day. There were three biological replicates.

### Sample and RNA Preparation

Female and male insects that were reared normally and treated with antibiotics for 15 days were washed with sterile water and then washed with 1% sodium hypochlorite, 75% alcohol, and sterile water for 1 min. In a disposable petri dish containing sterile water, the intestinal tract was dissected with sterile tweezers and Venus scissors. Each sample containing 10 intestinal tracts was immediately frozen in liquid nitrogen for 20 min and then transferred to −80°C for future use for intestinal tract transcriptome sequencing and analysis. Each sample containing one male and one female adult whole body was also immediately frozen in liquid nitrogen for 20 min and then transferred to −80°C for future use for whole-body transcriptome sequencing and analysis. Three biological repeats were prepared for transcriptome sequencing. RNA was extracted by an RNA simple Total RNA Kit (Tiangen, China) and sent to a company (BMK Biotechnology Co., Ltd., Beijing, China) for transcriptome sequencing.

### Isolation and Transcriptome Sequencing of Intestinal RNA

The whole genome of *B. dorsalis*, which was published in the National Center for Biotechnology Information (NCBI^1^) (PRJNA273958, ID: 10754), was used as a reference genome for the transcriptome assembly method. The raw RNA-Seq data has been deposited in the National Center for Biotechnology Information (NCBI) with accession code PRJNA694509 (ID: 694509). The sequencing data was obtained by constructing a transcriptome library (based on the Illumina HiSeq Platform). To verify the reliability of the data, three biological replicates were established for each treatment. For standardization, all paired reads from the clean data representing relative single gene expression levels were converted to fragments per kilobase per million mapped reads (FPKM) per thousand bases (Florea et al., 2013). Relative gene expression levels for each treatment were calculated using three repeated mean FPKMs. *p*-Values in multiple tests were corrected by a false discovery rate, according to Benjamini and Hochberg’s approach (Reiner et al., 2003). DEGs were identified based on a fold-change (FC) ≥ 2 and *p* < 0.01. Clean reads were aligned with the reference genome sequence using TopHat2 (Kim et al., 2013) to obtain genetic information, sequence features, and sample information. Gene Ontology (GO) enrichment analysis of the DEGs was implemented by the GOseq R package (V 1.16.2) based on Wallenius non-central hypergeometric distribution (Young et al., 2010). KEGG (Kanehisa et al., 2008) is a database resource for understanding the high-level functions and utilities of biological systems, such as cells, organisms, and ecosystems, from molecular-level information, especially large-scale molecular datasets generated by genome sequencing and other high-throughput experimental technologies^2^. KOBAS (Mao et al., 2005) software (3.0) was used to analyze the enrichment of DEGs in KEGG pathways. The sequences of the DEGs were blasted (blastx) to the genome of a related species [the protein--protein interaction (PPI) of which exists in the STRING database^3^ ] to obtain the predicted PPIs of these DEGs. Then, the PPIs of these DEGs were visualized in Cytoscape (Shannon, 2003).

### Quantitative Real-Time PCR

To verify the transcriptome data demonstrating *B. dorsalis* responding to changes in intestinal bacteria, 10 immunity-related genes were selected for quantitative real-time PCR (qRT-PCR) testing (Bai et al., 2018). Groups consisted of female and male insects with two types of controls and two types of treatments, and each group had three replicates (2 × 2 × 3 = 12). There were 12 groups and 10 intestines in each group. RNA was extracted using an RNA simple Total RNA Kit (Tiangen, China). The RNA was then reverse transcribed into cDNA by RT-PCR using the PrimeScript RT Reagent Kit (Takara, Beijing). The resulting cDNA was stored at −20°C and diluted 10 times before being used. The cDNA template was amplified by PCR with GoTaq Green Master Mix (Tiangen, China) using primers for the α-tubulin gene for template detection.

For quantitative real-time PCR, the 18S rRNA gene was selected as the internal reference gene. Primers were designed by Premier 5.0, and specificity was verified by NCBI. The reaction system and reaction conditions for qRT-PCR were established following the instructions of GoTaq^®^ Green Master Mix (Tiangen, China). Reaction conditions were as follows: 95°C for 30 s; 95°C for 5 s, 60°C for 34 s (40 cycles); 95°C for 15 s, 60°C for 1 min, 95°C for 15 s, 60°C for 15 s. All primers were synthesized by Shanghai Bioengineering Company and their sequences are listed in Supplementary Table 1.

### Statistical Analysis

For each biological replicate, three technical replicates were performed. The relative expression level of genes was analyzed by the 2^–Δ^ ^Ct^ method (Chen and Wagner, 2012). The qRT-PCR results were statistically analyzed by Student’s *t*-tests using SPSS 22.0, and *p* < 0.05 was considered significant. Independent *t*-tests using SPSS 22.0 were used to test the growth, mortality, and reproduction of treated and untreated flies, and *p* < 0.05 was considered significant.

## Results

### Gut Bacterial Flora and Function Prediction

The gut bacterial flora was significantly changed with antibiotic feeding (Bai et al., 2018). Table 1 shows that the major bacterial genera changed from *Enterobacter* to *Bacillus* and *Lactococcus* in males, while in females, the major genus was *Lactococcus* instead of *Pseudomonas*. The main bacteria in males and females shifted from Gram-negative bacteria to Gram-positive bacteria.

**TABLE 1:** Top 10 bacterial genera in the *Bactrocera dorsalis* control and antibiotic treatment groups.

**Bacteria (Genus)**	**Gram^1^**	**CKM**	**TRM**	**CKF**	**TRF**
*Enterobacter*	G−	72.49	0.09	6.23	2.82
*Pseudomonas*	G−	6.45	5.42	83.07	29.04
*Bacillus*	G+	0.01	43.34	0.01	5.71
*Lactococcus*	G+	1.03	32.98	4.78	33.86
*Stenotrophomonas*	G−	0.06	0.46	0	15.8
*Acinetobacter*	G−	16.27	11.19	0	7.77
*Leclercia*	G−	0.2	0	5.27	0
*Achromobacter*	G−	3.44	0	0	0
*Streptococcus*	G+	0	3.79	0	2.61
*Lactobacillus*	G+	0	0	0	0.13

*^1^Gram stain; G+, Gram positive; G–, Gram negative.*

*CKM, male in the control group; TRM, male intestinal tract in the antibiotic treatment group; CKF, female in the control group; TRF, female in the antibiotic treatment group.*

Bacterial function prediction showed that E (amino acid transport and metabolism), R (general function prediction only), and S (function unknown) were the top three important gene functions in adults (Supplementary Figure 1). In females, genes related to L (replication, recombination, and repair), A (RNA processing and modification), B (chromatin structure and dynamics), E (amino acid transport and metabolism), I (lipid transport and metabolism), K (translation), N (cell motility), Q (secondary metabolite biosynthesis transport and catabolism), and U (intracellular trafficking, secretion, and vesicular transport) decreased. By contrast, in males, genes related to A (RNA processing and modification), B (chromatin structure and dynamics), and K (translation) increased, while genes related to N (cell motility), U (intracellular trafficking, secretion, and vesicular transport), and W (extracellular structures) decreased.

### Growth and Reproduction

**FIGURE 1:**
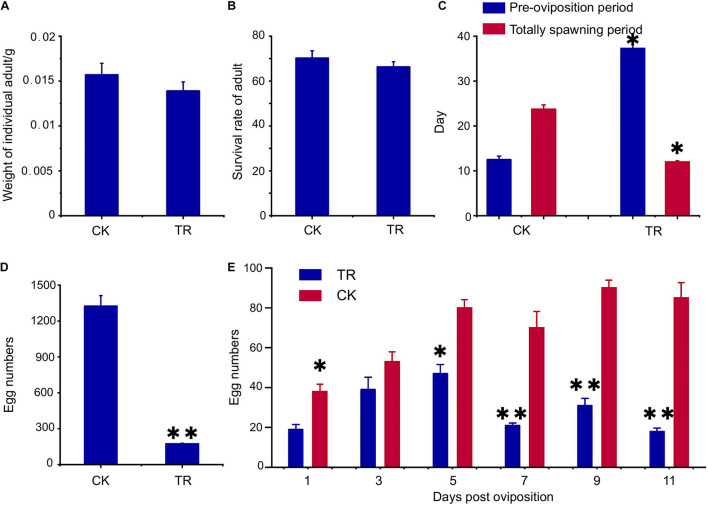
Antibiotic feeding showed different effects on the growth and reproduction of *Bactrocera dorsalis*. **(A)** Antibiotic feeding did not decrease the weight of adults; **(B)** antibiotic feeding did not decrease the survival rate of adults; **(C)** antibiotic feeding prolonged the preoviposition period and shortened the total spawning period; **(D)** antibiotic feeding decreased the total number of eggs; **(E)** antibiotic feeding decreased the number of eggs per day after oviposition. CK, fruit flies reared under the normal conditions; TR, treatment group fed antibiotics. *p < 0.05; **p < 0.01.

The effect of gut bacteria on host oriental fruit flies was also investigated by testing two growth and four reproductive factors after the gut bacterial diversity was changed using antibiotics; the results are shown in Figure 1. Growth factors, including adult weight (*p* = 0.38) and survival rate (*p* = 0.89), were not significantly changed by gut bacterial changes (Figures 1A,B). However, the preoviposition period was significantly prolonged, increasing from 12.5 to 23.7 days (*p* < 0.05) (Figure 1C). Additionally, females in the control group could produce offspring for more than 37 days, while the females in the treatment group had only 12 days of spawning. The spawning period was shortened significantly (*p* < 0.05) (Figure 1C). In addition, the total egg number (*p* < 0.01) and the egg number per day of females in the treatment group decreased significantly compared with the control group (Figures 1D,E) (1, 5 days, *p* < 0.05; 7, 9, 11 days, *p* < 0.01).

### Sequencing Data Analysis

After sequencing quality control was conducted by removing reads with an adapter, with null base and with low quality, a total of 95.60 GB of clean reads were obtained from 12 intestinal tract samples, and a total of 41.06 GB of clean reads were obtained from six adult whole-body samples. For each sample, no less than 7.05 GB of clean reads were obtained, and the percentage of Q30 bases for all the samples was more than 93.59%. Statistics of intestinal sequencing information are provided in Supplementary Table 2. The FPKM distribution of each sample is shown in Supplementary Figures 2A,C; this measured the expression level of each sample from the overall discrete angle of expression. The box plot shows that the overall gene expression levels of the different samples are similar. The correlation statistics between samples were plotted (Supplementary Figures 2B,D); the Pearson’s correlation coefficients between the same samples were 1 and within the same groups were close to 1. No abnormal samples were found. These results suggested that sampling was reliable and suitable for further analysis. The RNA quality of CKM1, CKM2, CKM3, CKF2, CKF3, TRM1, TRM2, TRM3, TRF1, TRF2, and TRF3 was high enough for further analysis, while the RNA quality of CKF1 only reached level B; this resulted in only 11 samples being used for the intestinal tract studies.

### Differentially Expressed Genes

A Venn diagram shows the differentially expressed genes (DEGs) shared between the groups (Supplementary Figure 2). In the adult whole body, there were 1,419 upregulated and 967 downregulated genes (Supplementary Figure 3A). In the male intestinal tract, there were 212 upregulated genes and 94 downregulated genes (Supplementary Figure 3B). In the female intestinal tract, 114 genes were upregulated and 168 genes were downregulated (Supplementary Figure 3C). There were also some non-differentially expressed genes in both males and females.

### Functional Annotation and Enrichment Analysis of Differentially Expressed Genes

Gene ontology analyses can define and describe genes and proteins to clarify the function of each gene. The GO annotation system consists of three major components, namely, biological process, molecular function, and cellular component, and 8,186 sequences were classified into 58 functional groups (Supplementary Figure 4). In the biological process category, 5,329, 5,316, and 4,470 genes were enriched for the terms single-organism process, cell process, and metabolic process, respectively. In the molecular functions category, 3,912 and 3,497 genes were annotated with the terms binding and catalytic activity, respectively. The cell and cell parts were dominant terms in the cellular component category, and 4,350 and 4,353 genes were enriched, respectively. The top 10 GO terms were single-organism process, cellular process, metabolic process, cell part, cell, binding, catalytic activity, organelle, biological regulation, and developmental processing. In total, 5,404 genes were successfully annotated by COG and were classified into 25 COG groups. Most genes were enriched in general functional prediction (24.37%), followed by replication, recombination and repair (7.59%), transcription (7.44%), amino acid transport and metabolism (6.72%) and posttranslational modification, protein turnover, and chaperones (6.35%). We speculated that there may be some new unknown genes under the unknown function term (2.35%).

### Functional Annotation and Enrichment Analysis of Adult Whole-Body Differentially Expressed Genes

**FIGURE 2:**
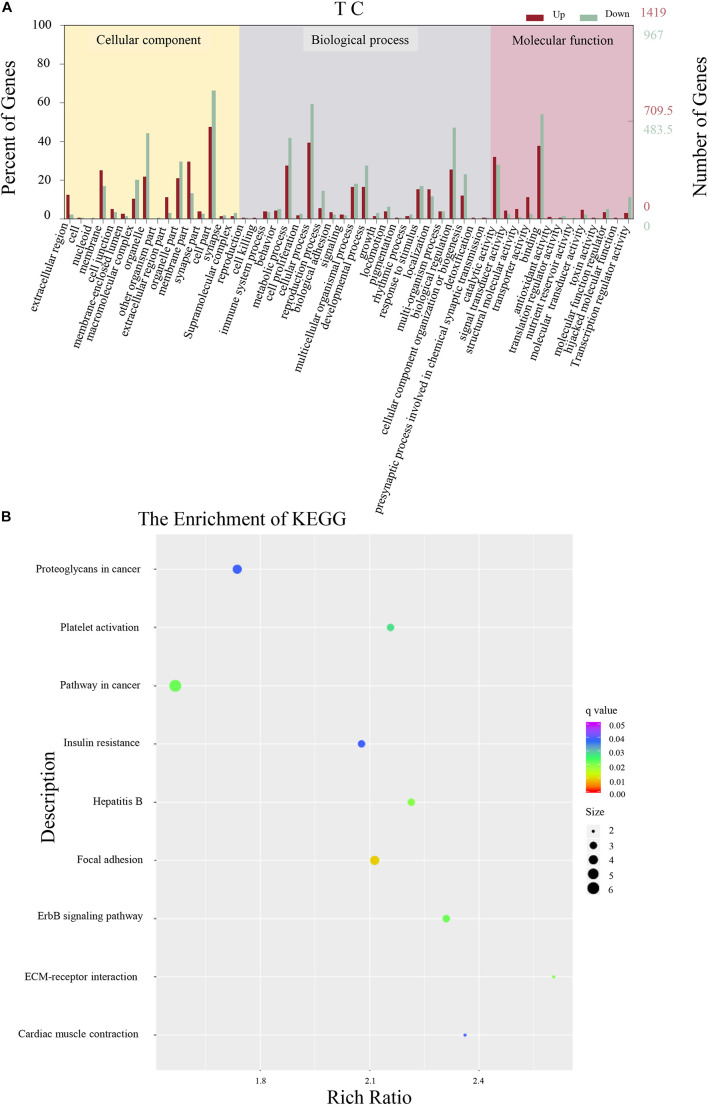
Differentially expressed gene analysis of adult whole-body samples. **(A)** Annotated statistical graph of the Gene Ontology (GO) secondary nodes. Light colors represent all genes and dark colors represent all differentially expressed genes (DEGs) of *Bactrocera dorsalis* adults. **(B)** Scatter plot of enriched KEGG pathways. Each circle in the graph represents the number of genes enriched for a specific KEGG pathway. Enrichment factors represent the ratio of the number of differentially expressed genes to all the genes in the pathway.

A total of 12,610 DEGs were identified in antibiotic females (Figure 2A). Among the 4,656 secondary nodes related to cellular components, 5,524 and 2,430 genes were enriched for biological processes and molecular functions, respectively. Among the cellular components, the cell parts term was the most abundant and had 1,306 genes. In the secondary nodes related to biological processes and molecular functions, cellular process and binding were the most abundant terms and had 1,127 and 1,049 genes, respectively. All unigenes from antibiotic females were enriched in a total of 304 KEGG metabolic pathways. The top nine KEGG pathways are shown in Figure 2B. Among them, the most highly enriched factor was in the pathway named cardiac muscle contraction (KO04260), and the most DEGs were enriched for the term pathways in cancer (KO05200).

### Functional Annotation and Enrichment Analysis of Male Intestinal Tract Differentially Expressed Genes

**FIGURE 3:**
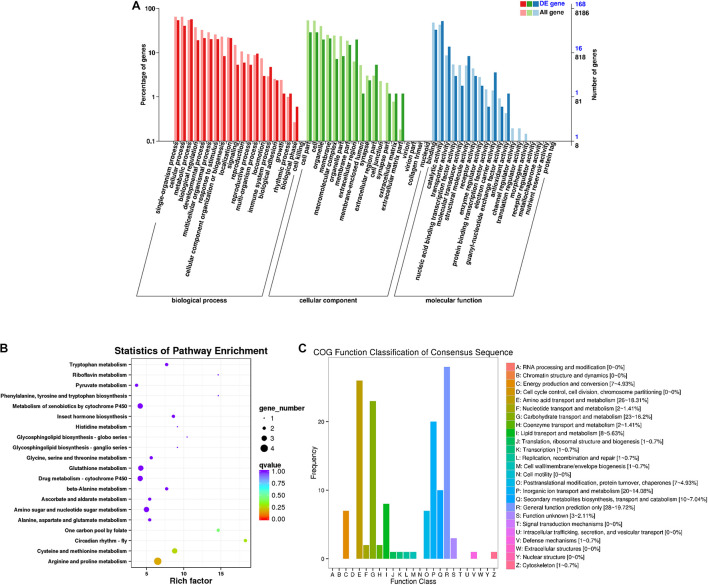
Male differentially expressed gene analysis. **(A)** Annotated statistical graph of the GO secondary nodes. Light colors represent all genes and dark colors represent all differentially expressed genes (DEGs) of adult males. **(B)** Scatter plot of enriched KEGG pathways. Each circle in the graph represents the number of genes enriched for a specific KEGG pathway. Enrichment factors represent the ratio of the number of differentially expressed genes to all the genes in the pathway. **(C)** COG annotation classification statistics of differentially expressed genes in males. Frequency is the number of genes.

A total of 168 DEGs were identified in males (Figure 3A). Among the secondary nodes related to biological processes, 96, 91, and 68 genes were enriched for metabolic processes, single-organism processes, and cellular processes, respectively. Among the cellular components, cells and cell parts were the most abundant and had 48 genes. In the secondary nodes related to molecular function, catalytic activity and binding activity were the most abundant terms, with 87 and 55 genes, respectively. There were 142 differentially expressed genes in the 25 COG groups (Figure 3C). Among them, the most genes, with a percentage of 19.72% (28), were enriched for general functional prediction terms, followed by amino acid transport and metabolism and carbohydrate transport and metabolism, with percentages of 18.31% (26) and 16.2% (23), respectively. Three genes were enriched for unknown function items (2.21%). We speculated that these unknown sequences may contain some new unknown genes. All unigenes from males were enriched in a total of 58 KEGG metabolic pathways. The top 20 KEGG pathways are shown in Figure 3B. Among them, the most highly enriched factor was in the pathway named circadian rhythm-fly (KO04711), and most DEGs were enriched for the arginine and proline metabolism pathway (KO00330).

### Functional Annotation and Enrichment Analysis of Female Intestinal Tract Differentially Expressed Genes

**FIGURE 4:**
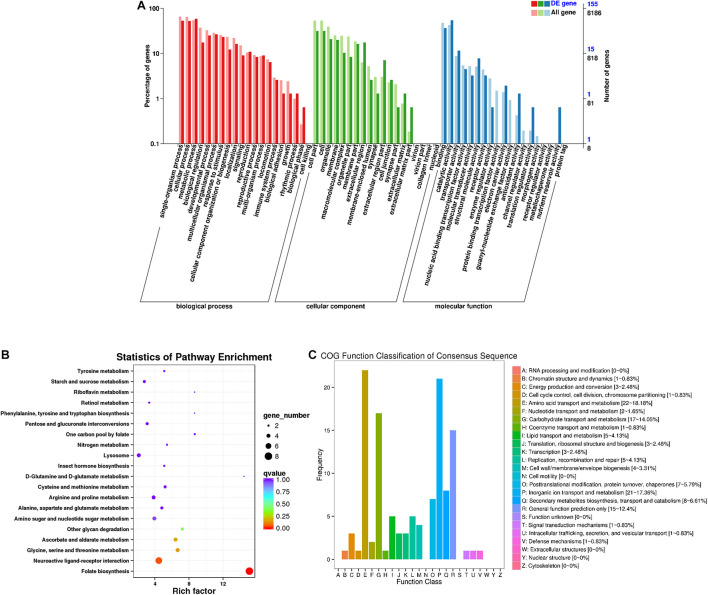
Female differentially expressed gene analysis. **(A)** Annotated statistical graph of GO secondary nodes. Light colors represent all genes and dark colors represent all differentially expressed genes in females. **(B)** Scatter plot of enriched KEGG pathways. Each circle in the graph represents the number of genes enriched for a specific KEGG pathway. Enrichment factors represent the ratio of the number of differentially expressed genes to all the genes in the pathway. **(C)** COG annotation classification statistics of differentially expressed genes in females. **(B)** Frequency is the number of genes.

A total of 155 DEGs were identified in females (Figure 4A). Among the secondary nodes related to biological function, 91, 82, and 81 genes were enriched for metabolic processes, single-organism processes, and cellular processes, and 81 genes were enriched for the secondary nodes related to cellular components. Among the secondary nodes related to molecular function, catalytic activity and binding activity were found to be the most abundant terms, with 84 and 57 genes, respectively. There were 121 differentially expressed genes in the 25 COG groups (Figure 4C). Amino acid transport and metabolism had the most genes, with a percentage of 18.18% (22), followed by inorganic ion transport and metabolism and carbohydrate transport and metabolism, with percentages of 17.36% (21) and 14.05% (17), respectively. No genes with unknown function were found. All unigenes from females were enriched in a total of 55 KEGG metabolic pathways. The top 20 KEGG pathways are shown in Figure 4B. The most highly enriched factor and the most differentially expressed genes were in the folate biosynthesis pathway (KO00790).

### Cluster Analysis of Differentially Expressed Genes Related to Immunity

Based on the KEGG pathway analysis of female adults 15 days post emergence of *B. dorsalis*, a total of 17 intestinal immune-related pathways were identified, including glycolysis, amino acid metabolism, pyruvate metabolism, and programmed cell death (Table 2). The results from male adults and adult whole-bodies (including females and males) were the same as those from females (Table 2). Ten immune-related genes were identified among the DEGs according to their RPFM values and gene function annotations; they are listed in Table 3. Among them, only three genes were speculated to play a role in males, including gram-negative bacteria-binding protein 3-like (*GNBP-3-like*), myb-like protein M (*MP-M*), and lysosome membrane protein 2 (*LMP-2*). Six genes were speculated to play a role in females, including *Laccase-1*, octopamine receptor beta-1R (*OR*β*-1R*), probable multidrug resistance-associated protein lethal (2) 03659 (*PMRAPL(2)03659*), ubiquitin-60S ribosomal protein L40 (*URP-L40*), lysozyme B-like (*LB-like)*, and cytosolic aminopeptidase-like (*CA-like*). One gene that was speculated to play a role in both males and females was peptidoglycan-recognition protein SC2 (*PGRP-SC2*). Based on the DEGs and KEGG pathway analysis, arginine and proline metabolism may be important pathways in the intestinal immune mechanisms of *B. dorsalis*.

**TABLE 2:** Immune-related KEGG pathways enriched in *Bactrocera dorsalis* adults.

**Number**	**Pathway name**
1	Pentose and glucuronate interconversions
2	Glycine, serine, and threonine metabolism
3	Pyruvate metabolism
4	Tryptophan metabolism
5	Metabolism of xenobiotics by cytochrome P450
6	Ascorbate and aldarate metabolism
7	Retinol metabolism
8	Cysteine and methionine metabolism
9	Phenylalanine, tyrosine, and tryptophan biosynthesis
10	Glycolysis/gluconeogenesis
11	Arginine and proline metabolism
12	Sphingolipid metabolism
13	Drug metabolism–cytochrome P450
14	Phagosome
15	Glycosphingolipid biosynthesis–globo series
16	ECM-receptor interaction
17	Drug metabolism–other enzymes

**TABLE 3:** Expression of immune-related genes and their RT-qPCR validation.

**Sex**	**Annotation**	**RNA-Seq**		**RT-qPCR**	
		**log_2_FC**	***p*-Value**	**log_2_FC**	***p*-Value**
Male	*GNBP-3-like*	2.2403	0.000	0.6906	0.034
	*MP-M*	2.2014	0.009	0.8030	0.010
	*LMP-2*	2.0973	0.007	0.6029	0.060
Female	*Laccase-1*	−2.8094	0.000	−0.7832	0.012
	*LB-like*	−3.2173	0.000	−0.0623	0.052
	*OR*β*-1R*	3.3143	0.030	0.2238	0.031
	*PMRAPL(2) 03659*	2.1459	0.000	0.0804	0.142
	*URP-L40*	2.9374	0.000	−0.2237	0.306
	*CA-like*	3.3494	0.006	0.9409	0.004
Male and female	*PGRP-SC2*	Male: −2.64887 Female: −3.4597	0.000 0.000	2.4323 1.6164	0.000 0.000

### Quantitative Real-Time-PCR Validation

To validate the sequencing quality, 10 immune-related genes were chosen for RT-qPCR. In the RT-qPCR assay, the expression of seven genes, *PGRP-SC2-M*, *PGRP-SC2-F*, *GNBP-3-like*, *MP-M*, *LMP-2*, *OR*β*-1R*, *PMRAPL(2)03659*, and *CA-like*, were increased, while *LB-like*, *URP-L40*, and *Laccase-1* were decreased. Compared with the RNA-Seq results, the expression of *PGRP-SC2* and *URP-L40* was different from that of the transcriptome.

## Discussion

Bacteria in the midgut of *B. dorsalis* help improve host insecticide resistance and environmental adaptation, regulate growth and development, and affect male mating selection (Damodaram et al., 2016; Cheng et al., 2017; Raza et al., 2020). Many reports have attempted to uncover the function and regulatory mechanism of symbiotic bacteria in host insects. Amino acids have been confirmed to be the main factor that regulates the growth, development, and reproduction of host insects (Hoedjes et al., 2017; Ricardo et al., 2017). *Riptortus pedestris* gut bacteria can mediate growth, ovary development, and egg numbers by regulating three proteins stored in the hemolymph (Lee et al., 2016). During that process, food also plays an important part. Together with essential amino acids, two important gut bacteria, *Acetobacter* and *Lactobacillus*, can increase the egg production of *Drosophila* by making host flies prefer to feed on yeast (Ricardo et al., 2017). In our study, the most important predicted function of the gut bacteria in oriental fruit flies was related to amino acid transport and metabolism, which was consistent with a previous report that amino acids may be important in regulating the growth and reproduction of host flies (Ricardo et al., 2017).

With the rapid development of high-throughput sequencing technology, RNA-Seq has become an important tool for transcriptome research (Wang et al., 2009). The genus *Bactrocera*, in which *B. dorsalis* belongs, is by far the most frequently identified genus. DEGs were identified according to the gene expression levels in the different samples, and functional annotations and enrichment analyses were also performed. Many reports have shown that the transcriptome can be used to uncover biological phenomes and mechanisms of molecular regulation in *B. dorsalis* (Gu et al., 2019, Guo et al., 2020). Gut bacteria can improve host fly fitness via gene overexpression. A good example is the gut symbiont *Citrobacter* sp., which can help its *B. dorsalis* host degrade trichlorphon via the expression of phosphatase hydrolase genes. High-throughput sequencing technology has become a useful tool to explore new genes in bacteria or genes in host insects that are associated with the function of bacteria. A comparative genomic analysis between the gut and wild *Citrobacter* strains showed that phosphatase hydrolase genes were highly expressed in gut *Citrobacter* when trichlorphon was present (Cheng et al., 2017). The pyroquinolinin-dependent alcohol dehydrogenase gene (*PQQ-ADH*) of symbiotic acetic acid bacteria in *Drosophila* can regulate the development rate, individual size, energy metabolism, and intestinal stem cell activity of the host insects via the insulin pathway (Shin et al., 2011).

In this study, DEGs were assigned to 58 functional subcategories within three main categories (biological processes, cellular components, and molecular function), and most of the enriched terms shared between males and females were related to metabolic processes, catalytic activity, and single-organism processes. For the COG database annotations, the largest proportion of genes in males (19.72%) were enriched for general functional prediction terms. In females, genes related to amino acid transport and metabolism were the most enriched, accounting for 18.18%. For the top 20 KEGG enrichment pathways, the most differentially expressed genes among males were related to arginine and proline metabolism (KO00330), and the most differentially expressed genes in females were related to folate biosynthesis (KO00790). Our result is consistent with one previous report that the immunity of the host *B. dorsalis* in response to low temperature stimulates the arginine and proline metabolism pathway, and that this is promoted by the gut microbiota (Raza et al., 2020). Folic acid is very important during the development of female ovaries (Song et al., 2014). Reports have also shown that folic acid can be synthesized by the intestinal microflora in animals (Hanson and Gregory, 2011). These reports provide a wealth of genetic information for our follow-up experiments and will help us uncover the function of intestinal bacteria and the folate biosynthesis pathway in *B. dorsalis*.

Immunity is important for insects. Both diet and vertically transmitted bacteria can influence the fitness and immunity of *B. dorsalis* (Hassan et al., 2020). However, insects have an effective gut defense system that maintains self-immunity, thereby preserving the balance among microorganisms in the gut and stabilizing the diversity in gut symbiotic bacteria. It has been reported that the immune deficiency (Imd) pathway and dual oxidase–reactive oxygen species (Duox-ROS) are the main regulatory pathways in *B. dorsalis* (Wang, 2015; Liu et al., 2017; Iatsenko et al., 2018). Four genes, *PGRP-LB*, *PGRP-SB*, *cecropin*, and *defensin*, were confirmed to be key genes in the Imd pathway. In this study, to investigate the effect of intestinal bacteria on the intestinal immunity of *B. dorsalis*, differentially expressed genes and KEGG signaling pathways related to intestinal immunity were defined. As mentioned earlier, the *PGRP-SC2* and *URP-L40* validation results are controversial. For *URP-L40*, the different expression trends can be attributed to the high *p*-value obtained; the *p*-value for the qRT-PCR was larger; therefore, the data are less credible. It has been reported that *PGRP-SC2* can be downregulated with the activation of the Imd pathway in flies (Bischoff et al., 2006). *PGRP-SC2* has been confirmed to play an important role in innate immunity, and its expression level increased with increasing bacterial concentrations after challenge by Gram-positive bacteria in *Artemia sinica* (Zhu et al., 2017). Transcripts from different PGRP genes have been identified in immune regulatory organs such as the fat body, gut, and hemocytes in *Drosophila* (Werner et al., 2000). In our research, Gram-positive bacteria increased after the insects were fed antibiotics, and the expression of the *PGRP-SC2* gene in *B. dorsalis* rose accordingly, which was consistent with our experimental results. The *PGRP-SC2* gene and the arginine–proline metabolism pathway were identified and speculated to play a key role in the intestinal immunity of the *Drosophila* host, which provides a theoretical basis for revealing the function of intestinal bacteria. An advanced team showed that the *PGRP-SC2* gene could inhibit the intestinal overreaction of *B. dorsalis* caused by *E. coli* and that it plays a negative role in immune regulation (Yao, 2017). The key point of this study should be the effect of the *PGRP-SC2* gene on the intestinal commensal bacterial community of *B. dorsalis* and the role of *PGRP-SC2* in the immunity of *B. dorsalis*, which is also the target of our future research.

## Data Availability Statement

RNA-seq raw data have been deposited in National Center for Biotechnology Information (NCBI) with accession code PRJNA694509 (ID: 694509).

## Author Contributions

LL conceived and designed the study. LZe, LZh, and ZB performed the experiments. JF, LZh, and LL wrote the article. ZL and LL modified the manuscript. All authors have read and agreed to the published version of the manuscript.

## Conflict of Interest

The authors declare that the research was conducted in the absence of any commercial or financial relationships that could be construed as a potential conflict of interest.

## Publisher’s Note

All claims expressed in this article are solely those of the authors and do not necessarily represent those of their affiliated organizations, or those of the publisher, the editors and the reviewers. Any product that may be evaluated in this article, or claim that may be made by its manufacturer, is not guaranteed or endorsed by the publisher.

## Footnotes

1
http://www.ncbi.nlm.nih.gov/

2
http://www.genome.jp/kegg/

3
http://string-db.org/

## Supplementary Material

Supplementary Figure 1 COG-based functional distribution histogram of gut bacteria from *Bactrocera dorsalis* before and after antibiotic feeding. CKM, male in the control group; TRM, male in the antibiotic treatment group; CKF, female in the control group; TRF, female in the antibiotic treatment group.

Supplementary Figure 2 Sample reliability verification diagram. **(A)** Box plot of the gene expression distribution of all intestinal tract samples. The abscissa represents different samples; the ordinate represents the logarithm of the sample expression FPKM. **(B)** Heatmap of the correlation in expression between two intestinal tract samples. **(C)** Box plot of the gene expression distribution of the adult whole-body samples. The abscissa represents different samples; **(D)** heatmap of the correlation in expression between two adult whole-body samples. The horizontal and vertical coordinates in the graph are sample numbers, and their order is determined by the results of correlation clustering. CKM, male intestinal tract in the control group; CKF, female intestinal tract in the control group; TRM, male intestinal tract in the antibiotic treatment group; TRF, female intestinal tract in the antibiotic treatment group. C, male and female whole-body in the control group; T, male and female whole-body in the antibiotic treatment group.

Supplementary Figure 3 Overall analysis of differentially expressed genes. **(A)** Volcano plot of differentially expressed genes in the adult whole-body samples from the control and treatment groups. Yellow dots show upregulated genes, blue dots show downregulated genes, and black dots show non-differentially expressed genes. **(B)** Volcano plot of differentially expressed genes in the intestinal tract of males from the control and treatment groups. **(C)** Volcano plot of differentially expressed genes in the intestinal tract of females from the control and treatment groups. Red dots show upregulated genes, green dots show downregulated genes, and black dots show non-differentially expressed genes.

Supplementary Figure 4 GO and COG DEG analysis results. **(A)** Statistical results of the GO classifications for all genes. Light colors represent all genes and dark colors represent all differentially expressed genes. **(B)** Statistical results of the COG classification for all genes. Frequency is the number of genes.

Supplementary Table 1 Primers used for real time RT-qPCR amplification.

Supplementary Table 2 Statistics of intestinal sequencing yield of *Bactrocera dorsalis*.
