# Supplementary material for: Comparative Transcriptomic Analyses of Antibiotic-Treated and Normally Reared Bactrocera dorsalis Reveals a Possible Gut Self-Immunity Mechanism
Source: Front Cell Dev Biol. 2021 Sep 21;9:647604. doi: 10.3389/fcell.2021.647604 (PMC8490719; doi:10.3389/fcell.2021.647604)
Supplement: Supplementary Figure 1 — COG-based functional distribution histogram of gut bacteria from Bactrocera dorsalis before and after antibiotic feeding. CKM, male in the control group; TRM, male in the antibiotic treatment group; CKF, female in the control group; TRF, female in the antibiotic treatment group. [file Data_Sheet_1.PDF]

Comparative transcriptomic analyses of antibiotic-treated and normally-reared *Bactrocera dorsalis* reveals a possible gut self-immunity mechanism

Jiajin Fu[1#], Lingyu Zeng[1#], Linyu Zheng[1#], Zhenzhen Bai[1], Zhihong Li[1], Lijun Liu[1*]

[1] College of Plant Protection, China Agricultural University, Beijing 100193, China;

*** Correspondence:**
Corresponding Author: Lijun Liu, College of Plant Protection, China Agricultural University, Beijing 100193, China. Tel: +86-10-62732068;

E-mail: ljliu@cau.edu.cn.

[#]: These authors contributed equally to this study.

Keywords: ***Bactrocera dorsalis*, intestinal bacteria, immunity, transcriptome, antibiotic treatment, *PGRP-SC2*.**

Attachment

Table S1. Primers used for real time RT-qPCR amplification.

Gene	Nucleotide sequence (5'-3') Upstream primers	Downstream primers	Product sequence (bp)
18s rRNA[51]	5'-GCGAGAGGTGAAATTCTTGG -3'	5'- CGGGTAAGCGACTGAGAGAG -3'	192
PGRP-SC2	5'-GCCGTTATCCATCACACCGCTG-3'	5'-GACCGTCACCACCGATCAAGAAG-3'	142
GNBP-3-like	5'-AACACCGTCACGCCGTAATGG-3'	5'-ATCGTCGCTCTACGCTCCACTT-3'	113
MP-M	5'-GCCACCAGCGAGTTACATACCAAT-3'	5'-CAATACCATCGGCTACAGGTCGTG-3'	92
LMP-2	5'-TGGTGAAGCTTGTAGAACGCATCTT-3'	5'-AGCATCATCCAAACAACTCGTCTCA-3'	121
LB-like	5'-GCTAATCACAGCGTGAGCTATGGA-3'	5'-CGGGAATCATCGGAGATTTCATCGT-3'	131
ORβ-1R	5'-CAGGAACGGCTTGTCTACAGGTC-3'	5'-ACTTGGATACTCGGTCGAGGCTT-3'	98
PMRAPL(2)03659	5'-GGACCGATCTCATTTGCGTGCTTTA-3'	5'-GTCACCGCTGTAGAAGTCGCTATTG-3'	83
Laccase-1	5'-CGTGCTTGCCGATGGTGTTGA-3'	5'-CCGTGCCAATGTATTGTTGTCTCCA-3'	147
URP-L40	5'-TCTACCATCCACCTTGTCCTCAGAC-3'	5'-GCAGTTGTACTTGCGAGCCAGTT-3'	87
CA-like	5'-ATATTGAAGGCTGGCGAATCGGTTT-3'	5'-CGTGAGCAAGTTGGACGGCATT-3'	89
α-Tubulin	5'-CGCATTCATGGTTGATAACG-3'	5'-GGGCACCAAGTTAGTCTGGA-3'	184

Table S2. Statistics of intestinal sequencing yield of *Bactrocera dorsalis*.

Samples	Clean reads	Clean bases	GC Content	%≥Q30
CKM1	28,549,877	8,534,184,412	40.49%	94.20%
CKM2	28,862,225	8,643,187,300	40.22%	94.66%
CKM3	23,569,850	7,050,128,244	39.45%	94.12%
CKF1	23,836,763	7,133,569,326	41.20%	94.58%
CKF2	26,109,443	7,808,829,488	41.79%	93.88%
CKF3	27,183,688	8,125,130,480	41.11%	93.69%
TRM1	26,769,270	7,993,737,412	41.49%	93.75%
TRM2	27,393,276	8,190,761,996	41.21%	93.59%
TRM3	30,144,957	9,026,385,002	42.17%	94.41%
TRF1	26,440,803	7,906,841,780	42.14%	95.36%
TRF2	23,825,644	7,117,556,628	42.94%	94.84%
TRF3	27,019,941	8,065,138,626	42.39%	94.94%
C1	44,596,564	6,689,484,600	41.29%	94.46%
C2	45,146,520	6,771,978,000	40.32%	94.38%
C3	45,719,898	6,857,984,700	41.97%	94.33%
T1	46,727,528	7,009,129,200	42.49%	94.18%
T2	44,555,690	6,683,353,500	42.37%	94.69%
T3	46,980,688	7,047,103,200	42.71%	94.6%

CKM: male intestinal tract in control group; CKF: female intestinal tract in control group; TRM: male intestinal tract in antibiotic treatment group; TRF: female intestinal tract in antibiotic treatment group; C: male and female whole-body in control group; T: male and female whole-body in antibiotic treatment group; Clean reads: the total number of paired end reads in the clean data; Clean bases: The total number of clean data bases; GC content: clean data GC content, that is, the percentage of G and C bases in the clean data; %≥Q30: percentage of bases with clean data mass values greater than or equal to 30.

Figure legends

Figure S1. COG-based functional distribution histogram of gut bacteria from *Bactrocera dorsalis* before and after antibiotic feeding. CKM: male in the control group; TRM: male in the antibiotic treatment group; CKF: female in the control group; TRF: female in the antibiotic treatment group.

Figure S2. Sample reliability verification diagram. A. Box plot of the gene expression distribution of all intestinal tract samples. The abscissa represents different samples; the ordinate represents the logarithm of the sample expression FPKM. B. Heatmap of the correlation in expression between two intestinal tract samples. C: Box plot of the gene expression distribution of the adult whole-body samples. The abscissa represents different samples; D: Heatmap of the correlation in expression between two adult whole-body samples. The horizontal and vertical coordinates in the graph are sample numbers, and their order is determined by the results of correlation clustering. CKM: male intestinal tract in the control group; CKF: female intestinal tract in the control group; TRM: male intestinal tract in the antibiotic treatment group; TRF: female intestinal tract in the antibiotic treatment group. C: male and female whole-body in the control group; T: male and female whole-body in the antibiotic treatment group

Figure S3. Overall Analysis of Differentially Expressed Genes. A: Volcano plot of differentially expressed genes in the adult whole-body samples from the control

and treatment groups. Yellow dots show upregulated genes, blue dots show downregulated genes, and black dots show nondifferentially expressed genes.

B: Volcano plot of differentially expressed genes in the intestinal tract of males from the control and treatment groups. C: Volcano plot of differentially expressed genes in the intestinal tract of females from the control and treatment groups. Red dots show upregulated genes, green dots show downregulated genes, and black dots show nondifferentially expressed genes.

Figure S4. GO and COG DEG analysis results. A: Statistical results of the GO classifications for all genes. Light colors represent all genes and dark colors represent all differentially expressed genes. B: Statistical results of the COG classification for all genes. Frequency is the number of genes.

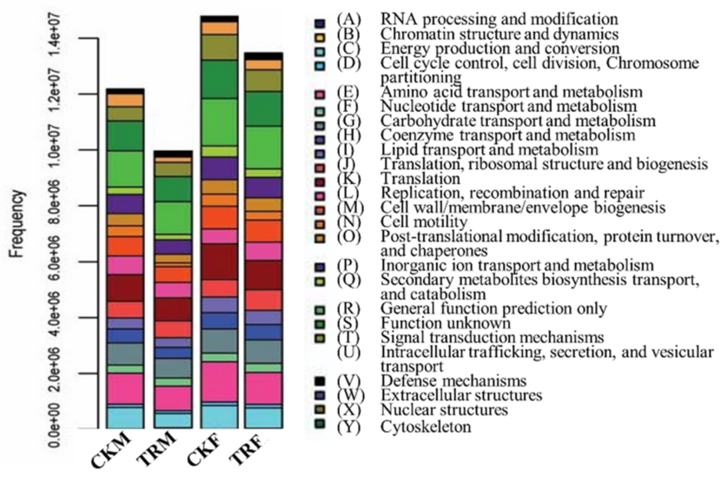

Figure S1. COG-based functional distribution histogram of gut bacteria from *Bactrocera dorsalis* before and after antibiotic feeding. CKM: male in the control group; TRM: male in the antibiotic treatment group; CKF: female in the control group; TRF: female in the antibiotic treatment group.

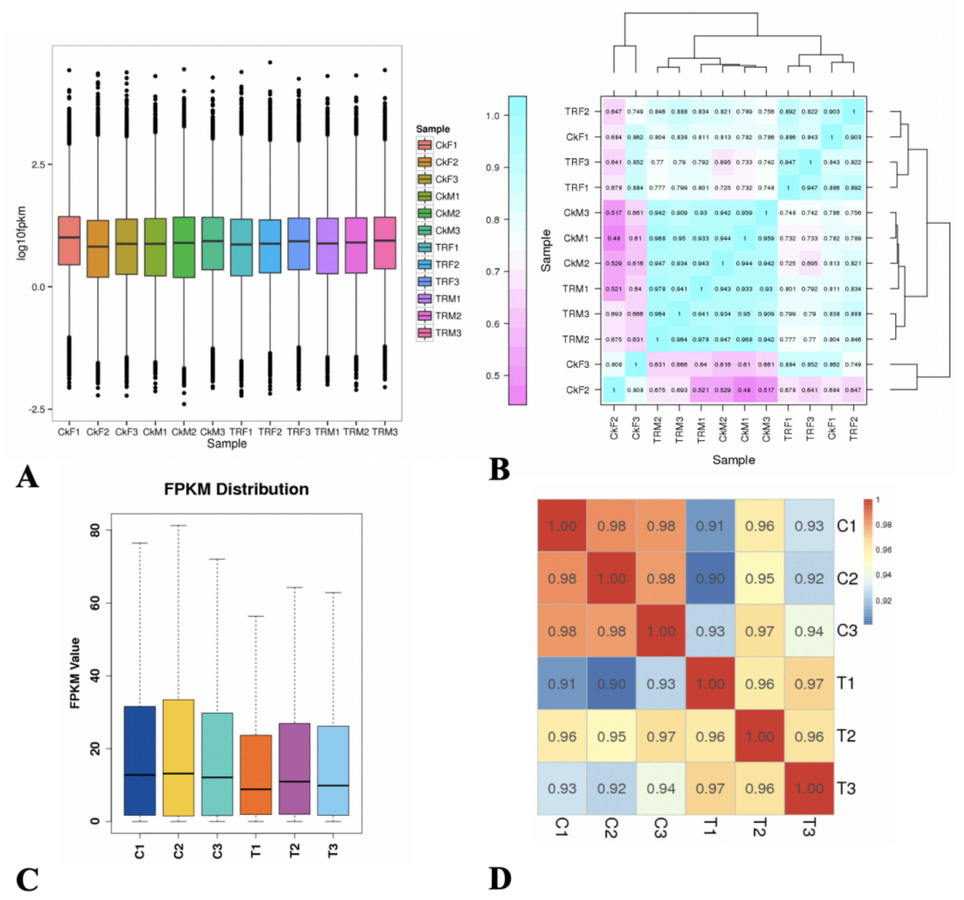

Figure S2. Sample reliability verification diagram. A. Box plot of the gene expression distribution of all intestinal tract samples. The abscissa represents different samples; the ordinate represents the logarithm of the sample expression FPKM. B. Heatmap of the correlation in expression between two intestinal tract samples. C: Box plot of the gene expression distribution of the adult whole-body samples. The abscissa represents different samples; D: Heatmap of the correlation in expression between two adult whole-body samples. The horizontal and vertical coordinates in the graph are sample numbers, and their order is determined by the results of correlation clustering. CKM: male intestinal tract in the control group; CKF: female intestinal tract in the control group; TRM: male intestinal tract in the

antibiotic treatment group; TRF: female intestinal tract in the antibiotic treatment group. C: male and female whole-body in the control group; T: male and female whole-body in the antibiotic treatment group

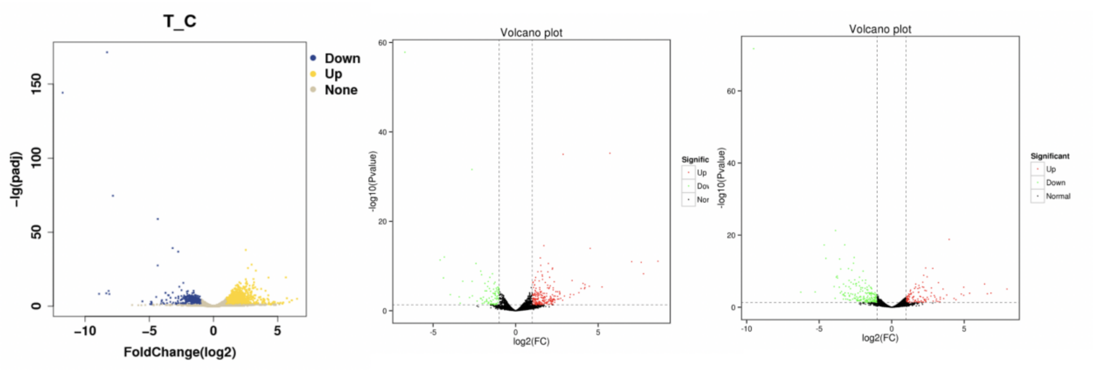

A **B** **C**

Figure S3. Overall Analysis of Differentially Expressed Genes. A: Volcano plot of differentially expressed genes in the adult whole-body samples from the control and treatment groups. Yellow dots show upregulated genes, blue dots show downregulated genes, and black dots show nondifferentially expressed genes.

B: Volcano plot of differentially expressed genes in the intestinal tract of males from the control and treatment groups. C: Volcano plot of differentially expressed genes in the intestinal tract of females from the control and treatment groups. Red dots show upregulated genes, green dots show downregulated genes, and black dots show nondifferentially expressed genes.

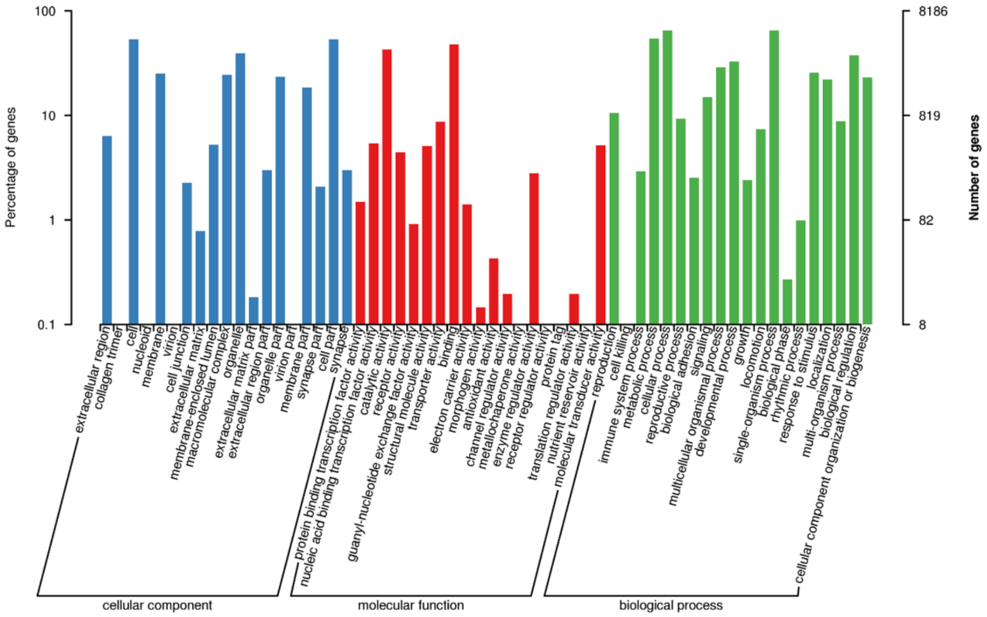

A

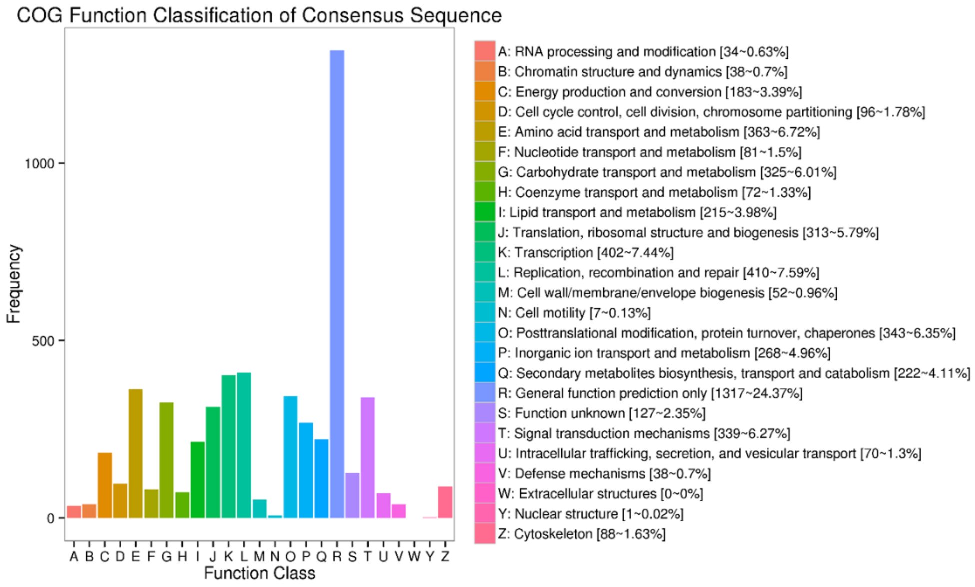

B

Figure S4. GO and COG DEG analysis results. A: Statistical results of the GO classification for all genes. Light colors represent all genes and dark colors represent all differentially expressed genes. B: Statistical results of the COG classification for all genes. Frequency is the number of genes.
